# Personalized Mortality Risk Stratification in ALD- and MASLD-Related Hepatocellular Carcinoma Using a Machine Learning Approach

**DOI:** 10.3390/metabo16010008

**Published:** 2025-12-22

**Authors:** Miguel Suárez, Sergio Gil-Rojas, Pablo Martínez-Blanco, Ana M. Torres, Natalia Martínez-García, Miguel Torralba, Jorge Mateo

**Affiliations:** 1 Gastroenterology Department, Virgen de la Luz Hospital, 16002 Cuenca, Spain; 2 Medical Analysis Expert Group, Instituto de Investigación Sanitaria de Castilla-La Mancha (IDISCAM), 45071 Toledo, Spain; 3 Medical Analysis Expert Group, Institute of Technology, Universidad de Castilla-La Mancha, 16071 Cuenca, Spain; 4 Internal Medicine Unit, University Hospital of Guadalajara, 19002 Guadalajara, Spain; 5 Faculty of Medicine, Universidad de Alcalá de Henares, 28801 Alcalá de Henares, Spain; 6 Translational Research Group in Cellular Immunology (GITIC), Instituto de Investigación Sanitaria de Castilla-La Mancha (IDISCAM), 45071 Toledo, Spain

**Keywords:** hepatocellular carcinoma, machine learning, Random Forest, alcohol-associated liver disease, metabolic-dysfunction associated steatotic liver disease, prognosis, Artificial Intelligence

## Abstract

**Background/Objectives:** The epidemiology of hepatocellular carcinoma (HCC) is shifting, with alcohol-associated liver disease (ALD) and metabolic dysfunction-associated steatotic liver disease (MASLD) becoming leading causes in developed countries. This study aimed to identify the main prognostic factors for mortality at diagnosis in HCC patients with ALD and MASLD using machine learning (ML) algorithms. Random Forest (RF) was proposed as reference method. **Methods:** A multicenter, retrospective cohort of 91 patients diagnosed with HCC due to ALD or MASLD between 2008 and 2023 was analyzed. Demographic, clinical, and biochemical variables were collected. Several ML algorithms were implemented: RF, Support Vector Machine, Decision Tree, Gaussian Naïve Bayes, and K-Nearest Neighbors. Bayesian optimization was applied for hyperparameter tuning. Model performance was evaluated using standard metrics including AUC, precision, recall, and F1 score. **Results:** RF achieved the highest performance across all metrics (AUC: 0.91, precision: 90.67%, F1 score: 91.05%), surpassing other algorithms by over 10%. The most relevant variables for mortality prediction were serum albumin, CRP/albumin ratio, BCLC stage, and ALBI score. MELD 3.0 showed superior predictive value compared to other MELD variants. Conversely, AFP had limited prognostic utility in this population. **Conclusions:** In HCC patients related to ALD and MASLD, liver function and systemic inflammation markers outperform tumor markers for early mortality prediction. In this cohort, RF offered the highest predictive performance among the evaluated algorithms and may support personalized prognosis in ALD- and MASLD-related HCC; however, external validation in independent datasets is required before broad clinical implementation.

## 1. Introduction

Hepatocellular carcinoma (HCC) is the most common primary liver tumor, accounting for approximately 90% of primary liver cancers [1]. Among all malignancies, it ranks as the sixth most common cancer worldwide and the third leading cause of cancer-related death [2]. Its incidence and mortality are both on the rise. It is estimated that the number of annual cases will increase from around 900,000 in 2020 to approximately 1,400,000 by 2040. This rise is primarily driven by population aging, persistent exposure to risk factors, and the growing prevalence of chronic liver disease in both developed and developing countries [3].

The development of HCC is strongly associated with advanced chronic liver disease (ACLD), which confers an annual risk of up to 2% [4]. Historically, chronic hepatitis C virus (HCV) and hepatitis B virus (HBV) infections have been the predominant etiologies [5,6]. However, in recent years, alcohol-associated liver disease (ALD) and metabolic dysfunction-associated steatotic liver disease (MASLD) have emerged as major drivers of HCC [7]. MASLD, formerly known as non-alcoholic fatty liver disease (NAFLD), is now recognized as a major cause of chronic liver disease globally, paralleling the worldwide epidemics of obesity and metabolic syndrome. ALD, similarly, remains a persistent and growing public health burden, especially in high-income countries with increasing alcohol consumption trends [8,9,10].

The epidemiology of HCC is undergoing a substantial transition. In many regions, universal HBV vaccination and the introduction of highly effective antiviral therapies for HBV and HCV have led to a decline or stabilization of virus-related HCC incidence [11]. At the same time, MASLD and ALDs are increasing in prevalence, positioning both as the leading causes of HCC in the near future [11,12]. Also, genetic predisposition further modulates disease risk in these populations. Polymorphisms in genes such as PNPLA3 (I148M), TM6SF2 (E167K), and HSD17B13 have been associated with increased susceptibility to fibrosis progression and HCC development in MASLD. This highlights the complexity and heterogeneity of MASLD-related HCC [13].

HCC arising from MASLD and ALD frequently presents with distinct biological and clinical features compared to viral etiologies. These patients often have lower alpha-fetoprotein (AFP) secretion, more advanced tumor stages at diagnosis, higher levels of systemic inflammation, and a greater burden of metabolic comorbidities [14,15]. Moreover, these tumors are frequently diagnosed outside of established screening programs, reducing the likelihood of early-stage detection. This creates a substantial clinical challenge, as existing prognostic models, largely developed in viral HCC populations, may not adequately capture the unique risk profiles of MASLD- and ALD-related HCC [16,17].

Identifying reliable prognostic factors at the time of diagnosis is clinically critical. Early mortality in HCC is often determined not only by tumor burden but also by the underlying hepatic functional reserve, systemic inflammation, and patient performance status. Accurate risk stratification at this early stage could support individualized therapeutic strategies, better resource allocation, and improved patient counseling. It could also provide a foundation for optimizing surveillance and treatment algorithms tailored to this growing subgroup of patients.

Machine learning (ML) approaches offer a promising framework to address these challenges. Unlike traditional statistical models, which rely on predefined linear assumptions, ML algorithms can detect complex, nonlinear, and high-dimensional relationships between demographic, clinical, biochemical, and tumor-related variables [18,19,20]. These capabilities make them well suited to model heterogeneous data such as that encountered in real-world hepatology. ML has been increasingly applied in liver diseases for early detection of fibrosis, noninvasive diagnosis, prognostication, and treatment outcome prediction in HCC [21,22,23,24]. Importantly, these methods can incorporate both liver function and systemic inflammation biomarkers, variables often overlooked or underweighted in conventional prognostic scores.

Among the various ML methods, the Random Forest (RF) algorithm has emerged as a particularly attractive option for clinical modeling. RF is robust to overfitting, can handle missing data and mixed variable types, and provides interpretable variable importance measures [25,26]. Its strong performance across diverse biomedical applications suggests that it may outperform traditional prognostic models in MASLD- and ALD-related HCC, where classical tumor markers have limited utility.

Therefore, the aim of this study was to identify the main risk factors associated with early mortality at diagnosis in patients with HCC secondary to ALD and MASLD, using ML-based predictive modeling. We hypothesized that markers of liver functional reserve and systemic inflammation, such as albumin levels and CRP/albumin ratio, would outperform classical tumor markers for early mortality prediction in this specific patient population. Furthermore, by comparing different ML algorithms with RF as the reference model, we sought to evaluate their predictive performance and potential applicability to routine clinical practice in this emerging and high-risk group of HCC patients.

## 2. Materials and Methods

This multicenter study was conducted at Virgen de la Luz Hospital in Cuenca and the University Hospital of Guadalajara, reference hospitals in their provinces. It had a retrospective design, with data collection spanning from January 2008 to December 2023. The study was approved by the Ethics Committee of the University Hospital of Guadalajara, which waived the requirement for informed consent due to the retrospective nature of the analysis.

Inclusion criteria were defined as patients over 18 years of age with a diagnosis of HCC. This diagnosis could be made either through typical imaging characteristics in patients with ACLD [27]; or via histological confirmation in cases of diagnostic uncertainty or in patients without ACLD [28]. Exclusion criteria included patients diagnosed at other institutions and those lacking the necessary clinical data in their medical records to fulfill the study variables.

Three categories of variables were collected and classified as follows:

‑ Demographic variables:

This group included age, sex, date of HCC diagnosis, date of death, or date of censoring (defined as the last outpatient visit for those still alive at the end of the study period). Data were also collected on the presence of hypertension [29], diabetes mellitus (DM) [30], dyslipidemia (DL) [31], active alcohol and tobacco use. The diagnosis of MASLD was based on current clinical guidelines (alcohol consumption < 140 g/week for women and <210 g/week for men) [32]. For alcohol consumption above these thresholds, diagnosis of ALD was established [33].

‑ Analytical variables:

Laboratory parameters were collected at the time of HCC diagnosis or within the first month after diagnosis. These included the white blood cell count (cells/mm^3^), focusing on neutrophils and total leukocytes; international normalized ratio (INR); platelet count (10^3^/dL); bilirubin (mg/dL); aspartate aminotransferase (AST, U/L); alanine aminotransferase (ALT, U/L); alkaline phosphatase (ALP, U/L); gamma-glutamyl transferase (GGT) (U/L); alpha-fetoprotein (AFP) (ng/mL); creatinine (mg/dL); albumin (g/dL); sodium (mEq/L); potassium (mEq/L); and C-reactive protein (CRP).

‑ Tumor-related variables

Patients included in the analysis had HCC secondary to ALD or MASLD. Collected tumor-related variables included BCLC stage [34], Eastern Cooperative Oncology Group (ECOG) stage [35], Child–Pugh score, Model for End-stage Liver Disease (MELD), MELD-Na, MELD 3.0 [36], TNM stage [37], Child–Pugh score [38], Albumin-bilirubin (ALBI) score [39], Fibrosis-4 (FIB-4) score [40], AST-to-Platelet Ratio Index (APRI) [41], presence of clinically significant portal hypertension (CSPH) [42], presence of ACLD [43], and if diagnosis was established in the screening program.

RF algorithm was proposed as the reference method for this study because of its well-documented strengths in handling complex and heterogeneous biomedical data. RF is an ensemble learning technique that constructs multiple decision trees on random subsets of both the data and predictor variables, combining their outputs through majority voting. This strategy substantially reduces variance and overfitting, which are common limitations of single-tree approaches. Moreover, RF naturally accommodates both numerical and categorical variables without requiring extensive preprocessing such as scaling or transformation, making it particularly suitable for real-world clinical datasets characterized by heterogeneity and noise [44]. While some studies have explored the application of ML in HCC, the number of publications focusing specifically on MASLD- and ALD-related HCC remains limited. To rigorously evaluate the discriminative performance of the proposed RF model, we compared it with four widely used ML algorithms representing different methodological families: Gaussian Naïve Bayes (GNB) [45], Decision Tree (DT) [46], Support Vector Machine (SVM) [47] y K-Nearest Neighbors (KNN) [48]. The resulting models were developed using MATLAB (The MathWorks, Natick, MA, USA; MATLAB.Version R2025a).

Continuous variables were standardized using z-score scaling for algorithms sensitive to feature magnitude (SVM and KNN). Tree-based models (RF and DT) and GNB were trained without scaling, as their performance is not affected by variable scale. To maximize predictive performance and prevent suboptimal parameterization, hyperparameters for each algorithm were tuned using Bayesian optimization. This sequential, model-based approach iteratively identifies the most promising hyperparameter configurations by learning from previous evaluations, thereby improving efficiency and reducing computational cost [49]. For RF, we optimized the number of trees (ranging from 50 to 500), minimum leaf size, maximum number of splits, and number of predictors sampled at each split. For SVM, kernel function (linear vs. Gaussian), box constraint, and kernel scale were tuned. KNN optimization included the number of neighbors and distance metric, while DT tuning focused on maximum splits, minimum leaf size, and split criterion (Gini vs. deviance). The following hyperparameter search spaces were defined:

- Random Forest (RF):
  - Number of trees: 50–500
  - Maximum number of splits: 10–200
  - Minimum leaf size: 1–20
  - Number of predictors sampled per split: 1–√p
- Support Vector Machine (SVM):
  - Kernel function: linear or RBF
  - Box constraint (C): 10^−3^ to 10^2^
  - Kernel scale (RBF): 10^−3^ to 10^2^
- k-Nearest Neighbors (KNN):
  - Number of neighbors (k): 1–30
  - Distance metric: Euclidean, cosine, or cityblock
  - Neighbor weighting: uniform or distance-weighted
- Decision Tree (DT):
  - Maximum number of splits: 10–200
  - Minimum leaf size: 1–20
  - Split criterion: Gini or deviance
- Gaussian Naïve Bayes (GNB):
  - Variance smoothing parameter: 10^−9^ to 10^−1^
  - Prior class probabilities: automatic or uniform

Feature importance for the RF was computed using the mean decrease in impurity (Gini importance). This method quantifies the contribution of each predictor to reducing node impurity across all splits in the ensemble, averaged over all trees. Higher values indicate variables that contribute more substantially to the model’s discriminatory performance.

**Figure 1:**
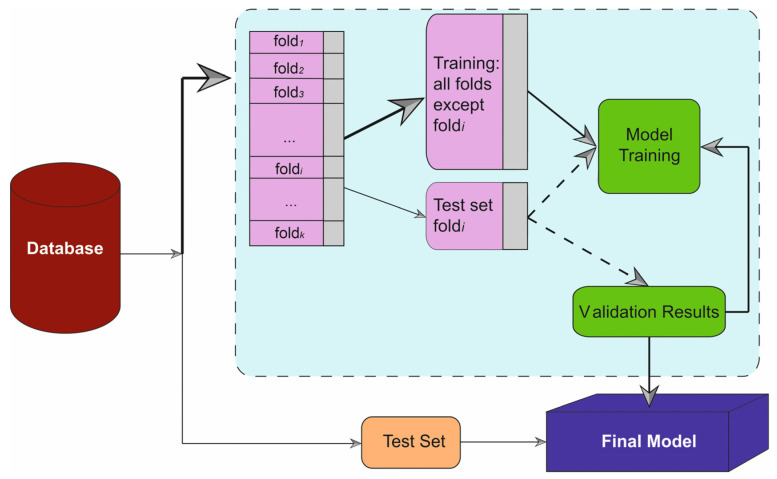
Graphical summary of the machine learning workflow used in this study. The dataset was divided into k folds for cross-validation. At each iteration, one fold (fold i) was used as the test set, while the remaining folds were used for model training. The ellipses (“…”) indicate intermediate folds omitted for graphical simplicity. Purple blocks represent data partitions (training and test folds), the green block represents the model training process, solid arrows indicate data flow during training, and dashed arrows indicate the testing phase.

Each optimization process was repeated 100 times to ensure stable mean and standard deviation estimates, thereby minimizing random variability and enhancing the reliability of model selection [50]. A five-fold cross-validation strategy was employed to mitigate overfitting, with the dataset divided into a training set (70%) and a testing set (30%), ensuring no overlap of patient data between phases to avoid leakage (Figure 1). Beyond discrimination, model calibration was evaluated using calibration plots and the Brier score. For each algorithm, predicted probabilities of early mortality were obtained on the independent test set. Patients were grouped into risk strata and observed event rates were plotted against mean predicted probabilities to generate calibration curves. Overall calibration performance was summarized using the Brier score, defined as the mean squared difference between predicted probabilities and the observed binary outcome (mortality vs. survival). Final model evaluation was performed on the independent test set, and all performance metrics were computed based on this validation procedure.

## 3. Results

**Figure 2:**
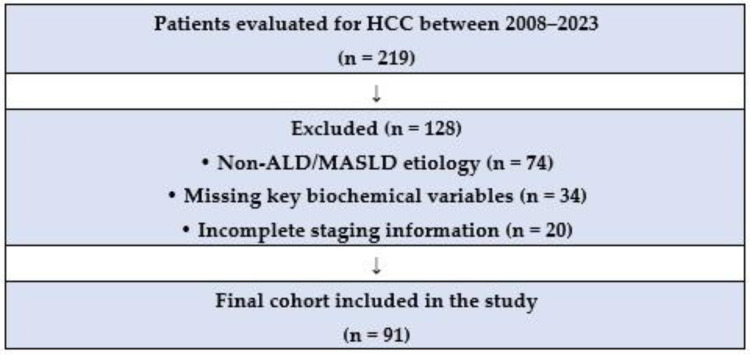
Flowchart summarizing the patient selection process for the study. ALD: Alcohol-associated Liver Disease. MASLD: Metabolic disfunction-Associated Steatotic Liver Disease.

Initially, a total of 219 patients were included in the study. Considering that the aim was to evaluate patients whose primary etiology of HCC was secondary to ALD or MASLD, 91 patients were ultimately analyzed. Of these, 68.13% belonged to the ALD group. Notably, alcohol was the leading cause of HCC in the entire cohort, surpassing HCV infection. A flow diagram summarizing the cohort selection process, including initial screening, exclusion criteria, and final sample size, has been added as Figure 2. In addition, Table 1 provides a summarized overview of the baseline characteristics of the 91 patients included in the study.

**Figure 3:**
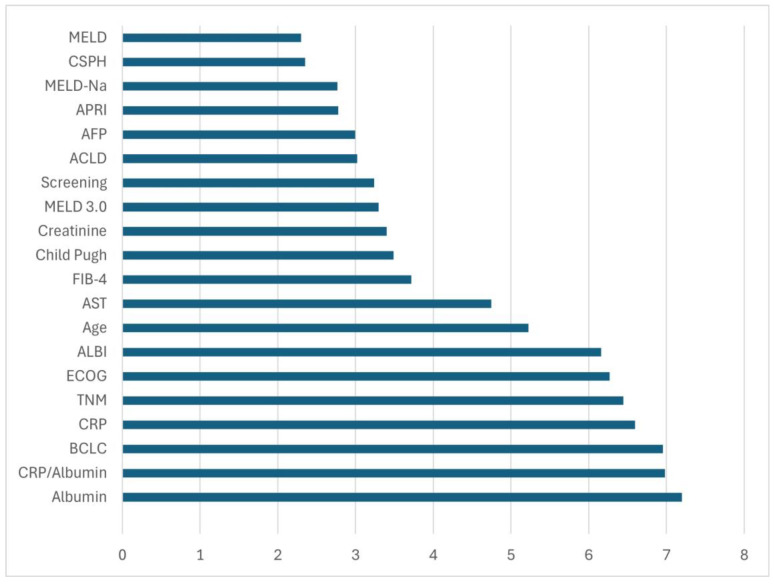
Final predictive model developed, showing the variables with the greatest relative weight. MELD: Model for End-stage Liver Disease; APRI: AST to Platelet Ratio Index; AFP: alpha-fetoprotein; FIB-4: Fibrosis-4; AST: Aspartate Aminotransferase; ALBI: Albumin-bilirubin score; ECOG: Eastern Cooperative Oncology Group stage; CRP: C-Reactive Protein; BCLC: Barcelona Clinic Liver Cancer stage.

Figure 3 displays the relative importance of each predictor to the final RF mortality prediction model. Serum albumin emerged as the most influential variable, followed closely by the CRP/albumin ratio and the BCLC staging system. These findings emphasize the central role of liver functional reserve and systemic inflammation in early mortality among patients with HCC arising from ALD and MASLD. Albumin levels reflect the synthetic capacity of the liver and are also influenced by systemic inflammatory activity, making them a sensitive indicator of both hepatic reserve and disease severity. Similarly, the CRP/albumin ratio has been recognized as a composite marker that captures both the inflammatory state and nutritional status, two key prognostic domains in advanced liver disease. The BCLC stage, TNM classification, ECOG performance status, and ALBI score also demonstrated substantial weight, highlighting the relevance of tumor burden, patient functional capacity, and liver function in prognostication.

The performance of the different algorithms used is presented in the subsequent tables. These metrics were selected as they are commonly employed in scientific literature. All performance metrics are presented together with their corresponding 95% confidence intervals to provide a more comprehensive evaluation of uncertainty. In the first table (Table 2), values for accuracy, recall, precision, and Area Under the Curve (AUC) are shown. As observed, RF algorithm outperforms all other implemented algorithms across these metrics. RF model exceeds SVM, DT, and GNB by nearly or more than 10%. The only algorithm approaching the performance of RF is KNN. However, while RF achieves accuracy and precision values of 91.32% and 90.67%, respectively, KNN scores 87.35% and 86.73%. RF is also the only algorithm to surpass 90% in AUC.

Table 3 presents the results for the F1 score, Youden’s Dependent Index (DYI), Matthews Correlation Coefficient (MCC), and Kappa metrics. In this case, the performance of the RF model again surpasses that of the other final models. For instance, the differences between RF and GNB, the algorithm with the poorest results, exceed 10% across all metrics. Focusing on MCC values, one of the most reliable indices due to its relationship with the four categories of the confusion matrix [51], RF also outperforms the other models. The MCC for RF was slightly above 80%, while for SVM, the third-best performing algorithm, it was 73.03%, a clearly significant difference in favor of RF.

**Figure 4:**
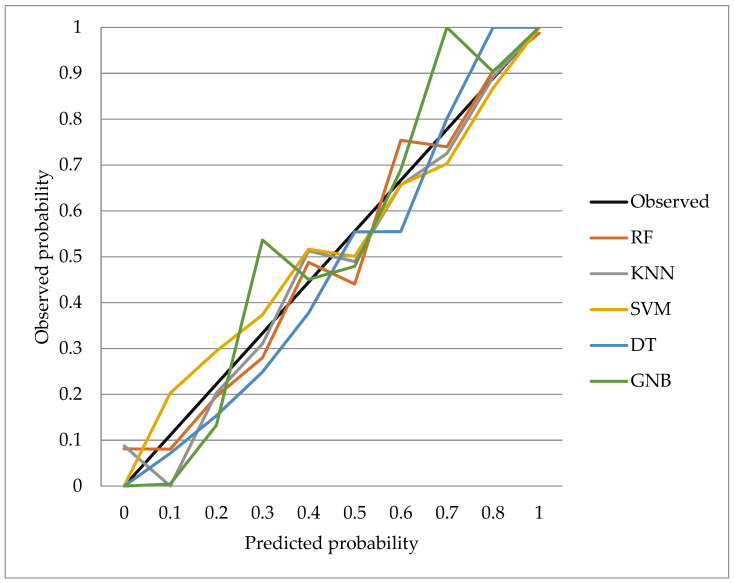
Calibration plots of all models. The RF model shows the best calibration with the lowest Brier score (0.13), while KNN, SVM, DT, and GNB exhibit progressively poorer agreement between predicted and observed mortality risk. RF: Random Forest; KNN: K-Nearest Neighbors; SVM: Support Vector Machine; DT: Decision Tree; GNB: Gaussian Naïve Bayes.

According to the calibration performance, the RF model achieved the lowest Brier score (0.13), indicating the best overall agreement between predicted and observed mortality risk, followed by KNN (0.15). SVM, DT, and GNB showed higher Brier scores (0.18, 0.19, and 0.21, respectively), consistent with their lower discriminative performance. Calibration plots for the test sets are shown in Figure 4 and confirm that RF maintains the closest alignment to the ideal diagonal, whereas the other algorithms tend to under- or overestimate risk in specific probability ranges.

**Figure 5:**
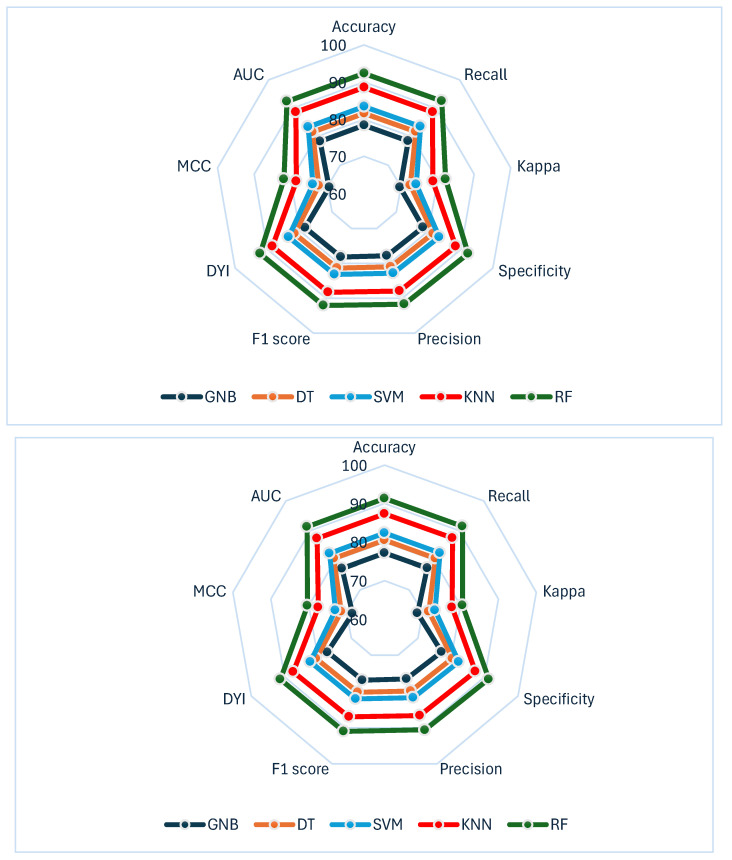
Summary of all analyzed metrics represented using a radar plot for all algorithms. The upper panel shows the training phase, while the lower panel depicts the test phase. AUC: Area Under the Curve; DYI: Youden’s Dependent Index; MCC: Matthews Correlation Coefficient; SVM: Support Vector Machine; DT: Decision Tree; GNB: Gaussian Naïve Bayes; KNN: K-Nearest Neighbors; RF: Random Forest.

To provide a comprehensive visualization of all performance metrics, a radar plot was generated (Figure 5). This figure integrates accuracy, recall, precision, AUC, specificity, F1 score, Youden’s Index, and MCC for each algorithm, enabling a global interpretation of their discriminative and calibration capacities. The upper panel corresponds to the training phase, whereas the lower panel depicts the test phase, allowing direct comparison between development and validation performance.

The RF model enclosed a nearly identical area in both phases, suggesting limited overfitting in internal validation. Nevertheless, these results derive from a single multicenter cohort, and the generalizability of the model to other populations remains to be established. This finding has important clinical implications. It indicates that the algorithm is able to maintain stable predictive performance when applied to new patients outside the training set, which is essential for any tool intended to support real-world clinical decision-making. In practice, this means that the probability of correct risk classification for an individual newly diagnosed with HCC of ALD or MASLD etiology would remain consistently high, irrespective of whether the patient belonged to the original training cohort.

Moreover, the RF model showed not only superior accuracy but also high MCC and specificity, which are key indicators of reliability in clinical predictive models. High specificity minimizes false-positive risk classifications, reducing unnecessary alarm or inappropriate escalation of care. Meanwhile, MCC value indicates that the RF model achieved balanced performance across all four categories of the confusion matrix (true positives, true negatives, false positives, and false negatives). This is particularly relevant in HCC, where misclassification of high-risk patients could delay life-saving interventions or, conversely, lead to overtreatment in lower-risk individuals.

In contrast, the other algorithms displayed smaller and less balanced enclosed areas between training and testing phases, suggesting lower robustness and generalization capacity. This reflects a higher susceptibility to performance drops when exposed to unseen data, which could limit their clinical applicability in dynamic and heterogeneous patient populations. The strong and stable performance of the RF model across all evaluated metrics positions RF as a promising tool for early risk stratification in this specific cohort. Future external and temporal validations are needed to confirm its performance and clinical applicability in other settings.

Taken together, these findings highlight the central role of hepatic functional reserve and systemic inflammation as major determinants of early mortality in patients with HCC secondary to ALD and MASLD. The predominance of albumin and CRP/albumin ratio over conventional tumor markers underscores that, in this specific population, host factors may be more decisive than tumor burden itself in shaping short-term prognosis. The strong and stable performance of the RF model, with high accuracy, specificity, and MCC across both training and internal validation phases, supports its potential utility as a risk stratification tool in patients with HCC secondary to ALD and MASLD. Such an approach could help clinicians identify high-risk patients at the time of diagnosis, allowing earlier referral to specialized centers, closer monitoring, and more aggressive therapeutic strategies when appropriate. In parallel, patients classified as lower risk could benefit from tailored follow-up strategies, potentially optimizing resource allocation and clinical outcomes. Nonetheless, external and temporal validation in larger, independent cohorts is required before considering it a clinically reliable tool for routine practice.

## 4. Discussion

Although globally HBV and HCV remain the leading causes of HCC due to their endemicity in Asia and sub-Saharan Africa, mainly reflecting limited vaccination programs and restricted access to antiviral therapy [1], a paradigm shift toward metabolic and alcohol-related etiologies is underway [52]. As antiviral coverage expands and universal HBV vaccination becomes more widespread, the incidence of viral-related HCC is expected to continue declining. In parallel, the global rise in obesity, the increasing prevalence of metabolic syndrome, particularly diabetes mellitus, and growing alcohol consumption are redefining the epidemiology of this tumor [53,54,55].

The recent redefinition of NAFLD to MASLD, along with the introduction of Metabolic dysfunction-associated alcohol-related liver disease (MetALD), reflects this clinical and biological transition. These new categories highlight the continuous and overlapping spectrum between metabolic dysfunction and alcohol consumption as synergistic risk factors for the development and progression of ACLD [32,56]. This interaction is particularly relevant because even moderate alcohol intake in patients with metabolic syndrome significantly increases the risk of developing HCC, often even in the absence of cirrhosis [21,57,58,59]. Importantly, the introduction of MetALD does not displace ALD as an etiology: chronic alcohol consumption, often underrecognized due to coexisting use disorders, continues to be one of the most important drivers of liver disease worldwide [33]. In the United States, ALD accounts for nearly 50% of hospital deaths related to cirrhosis and over 40% of liver transplants [60,61]. The steady increase in alcohol use, accelerated during the COVID-19 pandemic, underscores the urgency of implementing preventive strategies and optimizing risk stratification in these populations [62].

Our study highlights the prognostic significance of host- and liver-related factors in patients with HCC secondary to ALD and MASLD. Among all variables analyzed, serum albumin emerged as the strongest predictor of early mortality, closely followed by the CRP/albumin ratio, BCLC stage, and CRP itself. This pattern is particularly noteworthy, as tumor stage often dominates prognostic models, yet in this cohort markers of hepatic functional reserve and systemic inflammation outperformed traditional tumor characteristics [63,64]. Low albumin levels reflect not only impaired liver synthetic capacity but also the degree of systemic inflammation and nutritional status. Hypoalbuminemia has been repeatedly associated with worse outcomes in chronic liver disease, influencing both tumor progression and patient resilience to therapy [65,66,67]. Similarly, the CRP/albumin ratio integrates the systemic inflammatory response with nutritional and hepatic functional status [68,69,70], providing a composite marker that captures the multifactorial determinants of early mortality. The fact that these two variables rank at the top of the model emphasizes their potential clinical value for risk stratification at the time of diagnosis.

The ALBI score also demonstrated a strong predictive contribution, exceeding the performance of the Child–Pugh and MELD scores. Unlike Child-Pugh, ALBI is based exclusively on objective laboratory parameters, thereby eliminating the subjectivity associated with ascites or encephalopathy grading [71,72]. MELD scores, although valuable in many liver disease settings, often underestimate mortality in nonviral HCC, particularly in MASLD [73,74,75]. In our cohort, MELD 3.0 performed better than classical MELD and MELD-Na, likely because of the incorporation of albumin and sex into its calculation [76].While still inferior to albumin and CRP/albumin ratio, this finding suggests that MELD 3.0 may offer better risk stratification in patients with MASLD- and ALD-related HCC and warrants further validation in larger, external cohorts.

AFP, in contrast, was among the least relevant variables for mortality prediction at diagnosis. AFP levels in these patients may be confounded by protein-calorie malnutrition, sarcopenia, and chronic systemic inflammation, factors that are common in this population and can reduce marker sensitivity [77]. Furthermore, MASLD-related tumors frequently exhibit lower AFP production, diminishing its prognostic and diagnostic utility compared with viral HCC. This reinforces the need to investigate alternative noninvasive biomarkers and integrated models capable of more accurately reflecting disease biology in this group [78,79,80].

The application of ML in this context represents an important step toward more personalized prognostication. Unlike conventional statistical approaches, ML methods do not rely on linearity assumptions or variable independence. Instead, they learn directly from the data, identifying nonlinear interactions and complex patterns among clinical, biochemical, and tumor-related variables. This results in more flexible, data-driven predictive models with enhanced discrimination and calibration capabilities [81,82,83,84]. In this study, the RF algorithm was proposed as the reference method, given its excellent performance in heterogeneous clinical datasets. By combining multiple decision trees through bagging and random predictor selection, RF reduces variance, mitigates overfitting, and is robust to noise and missing data [85,86]. Moreover, its ability to output variable importance measures provides transparency and interpretability, key attributes for integrating predictive models into clinical workflows.

Our results show that RF outperformed SVM, DT, GNB, and KNN by more than 10 percentage points across several performance metrics, including AUC, F1, specificity, and MCC, while maintaining nearly identical performance between training and test phases. This stability is clinically relevant: it indicates that the model is capable of maintaining its predictive accuracy when applied to new patients, which is crucial for real-world applicability. A robust, interpretable ML model focusing on hepatic functional reserve and inflammation could support clinicians in early risk stratification, guiding therapeutic prioritization and follow-up intensity. In a setting where tumor burden is often not the primary prognostic driver, this approach could help identify patients who might benefit most from early intervention or closer monitoring.

Although the AUC was highlighted due to its strong performance, we acknowledge that the interpretation of ROC-based metrics may be influenced by outcome imbalance. In our cohort, the distribution of early mortality and survival showed only a modest class imbalance, which limits this concern. Precision–recall curves, typically more informative under severe imbalance, were therefore not included. Instead, to enhance transparency and reliability, we report 95% confidence intervals for all metrics, which are now incorporated in the updated tables.

This study has several limitations that should be acknowledged. A key limitation of this work is the relatively modest sample size, which reflects the real-world prevalence and availability of well-characterized ALD/MASLD-related HCC cases in our region. Although the cohort was multicenter, larger regional or national datasets would allow more extensive external validation and potentially improve model generalizability. The restriction to two centers within the same regional healthcare system may limit the representativeness of our cohort. Second, although we employed a rigorous internal validation strategy using 5-fold cross-validation and a 70/30 train–test split, we did not have access to an external cohort or to a sufficiently large dataset to perform a robust temporal validation (e.g., training on earlier years and testing on later years). Therefore, our findings should be considered hypothesis-generating, and the reported performance of the RF model may not directly extrapolate to other populations or healthcare settings.

Another limitation is the retrospective design of the study. Future studies using larger national or international registries, including external and temporal validation, are warranted to confirm the generalizability and clinical utility of the proposed model. It is necessary to add that our model was developed exclusively in patients with ALD- and MASLD-related HCC. Therefore, its performance in HCC secondary to viral hepatitis or other etiologies remains unknown. Comparative analyses across etiologies, ideally in independent external cohorts, are warranted to determine whether the same RF model can be generalized to non-ALD/MASLD HCC or whether etiology-specific models should be derived.

Finally, our retrospective dataset did not include standardized longitudinal follow-up, which precluded the construction of formal survival curves for albumin, CRP/albumin ratio, or other variables. Accordingly, the prognostic interpretation of these markers in our work is derived from their importance within the RF mortality-risk model at diagnosis and from previously published survival studies, rather than from time-to-event analyses performed in this cohort.

## 5. Conclusions

In patients with HCC secondary to MASLD and ALD, low serum albumin levels and an elevated CRP/albumin ratio are strong predictors of early mortality at diagnosis, reflecting the central prognostic role of hepatic functional reserve and systemic inflammation in this population, highlighting the predominant contribution of hepatic functional reserve and systemic inflammation in this setting. Rather than reflecting tumor burden alone, the model indicates that host-related factors play a central role in short-term outcome in ALD- and MASLD-related HCC. These results are in line with previous studies reporting that hypoalbuminemia and CRP-based indices are associated with adverse survival in patients with HCC.

Advanced disease stage within the BCLC classification is also closely associated with adverse outcomes, while the ALBI score demonstrated superior predictive value compared to other liver function scores. It is well established that advanced BCLC stage confers worse survival across all HCC etiologies; our findings emphasize that in ALD/MASLD-related HCC, hepatic functional reserve and systemic inflammation appear to be even more decisive than tumor burden itself in short-term mortality risk. Among MELD-based scores, MELD 3.0 showed better performance than conventional MELD and MELD-Na, suggesting that it may offer more accurate risk stratification in nonviral HCC.

Although it requires external validation, the RF algorithm achieved the highest overall predictive performance. This model outperforms other ML methods and demonstrates high stability between training and testing phases. This robustness highlights its potential as a clinically applicable tool for early risk stratification in patients with HCC related to MASLD and ALD. Integrating such predictive models into clinical workflows could help identify high-risk individuals at diagnosis, inform treatment prioritization, tailor follow-up intensity, and ultimately improve patient outcomes.

These findings underscore the need to look beyond tumor-related variables and incorporate host factors into prognostic assessment for this growing patient population. Future studies with larger, multicenter cohorts and external validation are warranted to confirm these results and facilitate the translation of predictive models into routine, data-driven clinical decision-making.

## Figures and Tables

**Table 1:** Baseline characteristics of patients with ALD- and MASLD-related HCC at diagnosis. CSPH: Clinically Significant Portal Hypertension. CRP: C-Reactive Protein.

Characteristic	Category/Unit	n (%) or Median (IQR)
Age at Diagnosis	Years	67.00 (60.00–74.00)
Overall Survival	Months	14.93 (6.77–25.10)
Demographics and Comorbidities		
Sex	Male	143 (79.0%)
	Female	38 (21.0%)
Alcohol Consumption	Yes	73 (40.3%)
Smoking Status	Smoker	82 (45.3%)
Diabetes Mellitus	Yes	72 (39.8%)
Obesity	Yes	33 (18.2%)
Dyslipidemia	Yes	67 (37.0%)
Hepatic Function and Disease Status		
Cirrhosis	Yes	155 (85.6%)
CSPH	Yes	22 (12.1%)
Ascites	Yes	33 (18.2%)
Encephalopathy	Yes	6 (3.3%)
MELD Score		9.00 (7.00–12.00)
Albumin	g/dL	3.50 (3.10–3.90)
INR		1.10 (1.00–1.20)
Sodium		137.00 (135.00–139.00)
Tumor Staging and Prognosis		
Portal Vein Thrombosis	Yes	57 (31.5%)
Metastasis	Yes	22 (12.1%)
ECOG Performance Status	0	121 (66.9%)
	1	48 (26.5%)
	2	12 (6.6%)
BCLC Stage	A	78 (43.1%)
	B	67 (37.0%)
	C	32 (17.7%)
	D	4 (2.2%)
Milan Criteria	Meets Criteria	78 (43.1%)
Diagnosis and Surveillance		
Diagnostic Method	Imaging/Clinical	164 (90.6%)
	Biopsy	17 (9.4%)
Surveillance/Screening	Yes	63 (34.8%)
Inflammatory Parameters		
CRP	mg/L	4.80 (1.70–14.60)
CRP/Albumin Ratio	Ratio (quantitative)	1.34 (0.44–4.09)

**Table 2:** Performance results of all analyzed algorithms for accuracy, recall, precision, and AUC. SVM: Support Vector Machine; DT: Decision Tree; GNB: Gaussian Naïve Bayes; KNN: K-Nearest Neighbors; RF: Random Forest; AUC: Area Under the Curve.

Methods	Accuracy (95% CI)	Recall (95% CI)	Precision (95% CI)	AUC (95% CI)
**SVM**	82.30% *(80.1–84.1)*	82.40% *(80.3–84.5)*	81.72% *(79.5–83.1)*	0.82 *(0.80–0.84)*
**DT**	80.49% *(78.2–82.6)*	80.59% *(78.3–82.2)*	79.92% *(77.6–82.1)*	0.80 *(0.78–0.82)*
**GNB**	77.15% *(74.9–79.3)*	77.24% *(75.0–79.4)*	76.60% *(74.3–78.5)*	0.77 *(0.75–0.79)*
**KNN**	87.35% *(85.2–88.3)*	87.45% *(85.3–88.7)*	86.73% *(84.6–88.4)*	0.87 *(0.85–0.89)*
**RF**	91.32% *(89.3–93.3)*	91.43% *(89.4–93.4)*	90.67% *(88.7–92.7)*	0.91 *(0.89–0.93)*

**Table 3:** Summary of the different performance metrics analyzed for the algorithms employed. SVM: Support Vector Machine; DT: Decision Tree; GNB: Gaussian Naïve Bayes; KNN: K-Nearest Neighbors; RF: Random Forest; DYI: Youden’s Dependent Index; MCC: Matthews Correlation Coefficient.

Methods	Kappa (95% CI)	Specificity (95% CI)	F1 Score (95% CI)	DYI (95% CI)	MCC (95% CI)
**SVM**	73.27% *(71.3–75.3)*	82.21% *(80.1–84.3)*	82.06% *(80.0–84.1)*	82.30% *(80.2–84.4)*	73.03% *(71.0–75.1)*
**DT**	71.66% *(69.6–73.7)*	80.40% *(78.3–82.5)*	80.25% *(78.1–82.4)*	80.49% *(78.3–82.6)*	71.42% *(69.3–73.6)*
**GNB**	68.68% *(66.7–70.7)*	77.06% *(74.9–79.2)*	76.92% *(74.8–79.0)*	77.15% *(75.0–79.4)*	68.46% *(66.4–70.5)*
**KNN**	77.76% *(75.7–79.8)*	87.25% *(85.1–89.4)*	87.09% *(84.9–89.3)*	87.35% *(85.2–89.4)*	77.51% *(75.4–79.6)*
**RF**	80.58% *(78.5–82.7)*	91.22% *(89.3–93.2)*	91.05% *(89.0–93.1)*	91.32% *(89.3–93.3)*	80.32% *(78.3–82.4)*

## Data Availability

The datasets used and/or analyzed during the present study are available from the corresponding author on reasonable request.

## Abbreviations

The following abbreviations are used in this manuscript:

AFP	Alpha-Fetoprotein
ALBI	Albumin-Bilirubin score
ALD	Alcohol-associated Liver Disease
ALT	Alanine Aminotransferase
APRI	AST to Platelet Ratio Index
AST	Aspartate Aminotransferase
AUC	Area Under the Curve
BCLC	Barcelona Clinic Liver Cancer
CRP	C Reactive Protein
CSPH	Clinically Significant Portal Hypertension
DM	Diabetes Mellitus
DL	Dyslipidemia
DT	Decision Tree
DYI	Youden’s Dependent Index
ECOG	Eastern Cooperative Oncology Group
FIB-4	Fibrosis-4 score
GNB	Gaussian Naïve Bayes
GGT	Gamma-Glutamyl Transferase
HBV	Hepatitis B Virus
HCC	Hepatocellular Carcinoma
HCV	Hepatitis C Virus
INR	International Normalized Ratio
KNN	K-Nearest Neighbors
MASLD	Metabolic Associated Steatotic Liver Disease
MCC	Matthews Correlation Coefficient
MELD	Model for End-stage Liver Disease
MetALD	Metabolic dysfunction-associated Alcohol-related Liver Disease
Na	Sodium
RF	Random Forest
SVM	Support Vector Machine
TNM	Tumor, Node, Metastasis staging
